# Thin-Shelled PEGylated Perfluorooctyl Bromide Nanocapsules for Tumor-Targeted Ultrasound Contrast Agent

**DOI:** 10.1155/2018/1725323

**Published:** 2018-11-01

**Authors:** Arifudin Achmad, Aiko Yamaguchi, Hirofumi Hanaoka, Yoshito Tsushima

**Affiliations:** ^1^Department of Diagnostic Radiology and Nuclear Medicine, Gunma University Graduate School of Medicine, Showa-machi 3-39-22, Maebashi, Gunma 3718511, Japan; ^2^Department of Nuclear Medicine and Molecular Imaging, Faculty of Medicine, Universitas Padjadjaran, Jl. Eijkman 38, Bandung 40161, Indonesia; ^3^Oncology and Stem Cell Working Group, Faculty of Medicine, Universitas Padjadjaran, Jl. Eijkman 38, Bandung 40161, Indonesia; ^4^Department of Bioimaging Information Analysis, Gunma University Graduate School of Medicine, Showa-machi 3-39-22, Maebashi, Gunma 3718511, Japan

## Abstract

Shell thickness determines the acoustic response of polymer-based perfluorooctyl bromide (PFOB) nanocapsule ultrasound contrast agents. PEGylation provides stealth property and arms for targeting moieties. We investigated a modulation in the polymer formulation of carboxy-terminated poly(d,l-lactide-co-glycolide) (PLGA) and poly(d,l-lactide-co-glycolide)-*block*-polyethylene glycol (PLGA-*b*-PEG) to produce thin-shelled PFOB nanocapsules while keeping its echogenicity, stealth property, and active targeting potential. Polymer formulation contains 40% PLGA-PEG that yields the PEGylated PFOB nanocapsules of approximately 150 nm size with average thickness-to-radius ratio down to 0.15, which adequately hindered phagocytosis. Functionalization with antibody enables *in vitro* tumor-specific targeting. Despite the acoustic response improvement, the *in vivo* tumor accumulation was inadequate to generate an observable acoustic response to the ultrasound power at the clinical level. The use of PLGA and PLGA-PEG polymer blend allows the production of thin-shelled PFOB nanocapsules with echogenicity improvement while maintaining its potential for specific targeting.

## 1. Introduction

Gas-lipid microbubbles as ultrasound contrast agents (UCAs) enable microvasculature visualization but are incapable of tumor molecular evaluation due to their inability to extravasate and poor stability. Nanometric UCAs may directly reach molecular targets in tumor cells by the enhanced permeation and retention (EPR) effect if they are stable and have targeting capability [1]. Regarding their nanoscale design, a precise balance has to be made between material selection and physicochemical properties, including morphology, stability, and acoustic response [2].

Perfluorooctyl bromide (PFOB), a biocompatible perfluorocarbon, provided echogenicity when used as a liquid core in a versatile design of poly(d,l-lactide-co-glycolide) (PLGA) polymer nanocapsule UCA [3–5]. However, the acoustic response of PFOB PLGA nanocapsules was deemed limited [4, 6], unless the nanocapsule is being concentrated and exposed to high-frequency ultrasound [7]. The acoustic response improvement might rely on nanocapsule compressibility, which is influenced by the polymer choice and shell thickness [8]. Shell thickness influences dilatational deformation and translational motion effects, both of which play roles in the acoustic behavior of PFOB PLGA nanocapsule [9–12].

Several approaches to obtain thin-shelled nanocapsules have been investigated. Reduction of PLGA amount relative to PFOB in the polymer formulation lowered thickness-to-radius (T/R) ratio and raised acoustic response of plain PFOB nanocapsules made by the emulsion evaporation method [4, 5]. However, this reduction strategy may not apply to every polymer [13–15]. Studies showed that PLGA is indispensable to compete with the surfactant stabilizing the emulsion while maintaining the wetting condition of PFOB during the organic solvent evaporation [15–17].

The final nanocapsule's core-shell morphology resulted from the intertwined relation between polymer viscosity, hydrophobicity, and its adsorption to the organic solvent-aqueous phase. Surface modification, furthermore, is inevitable if stealth property and targeting capability are simultaneously desired. Hence, the polymer selection and formulation is crucial for thin-shelled nanocapsule design. By far, surface modification with polyethylene glycol (PEG) remains the standard [18]. The sole use of PLGA-PEG polymer produced core-shell PFOB nanocapsules with prolonged *in vivo* circulation time [19, 20]. Although the polymer reduction strategy was incapable of scaling down shell thickness when only PLGA-PEG is used [21], a combination between PLGA and PLGA-PEG polymer has not yet particularly evaluated to obtain thin-shelled PFOB nanocapsules.

The blends of PLGA and PLGA-PEG polymer have been able to fine-tune the surface morphology of PFOB microcapsules while maintaining its core-shell structure [17]. In this study, we evaluated whether the blends of carboxy-terminated PLGA and PLGA-PEG can produce thin-shelled PEGylated PFOB nanocapsules for tumor-targeting UCAs. The varying amount of PLGA-PEG within the formulation was assessed to achieve optimum hindrance from phagocytosis. The functionalization of the PEG chains with monoclonal antibody cetuximab was also tested for active targeting of epidermal growth factor receptor- (EGFR-)positive tumor.

## 2. Materials and Methods

### 2.1. Materials

Carboxy-terminated PLGA Resomer RG 502 H (PLGA-COOH, lactic : glycolic acid 50 : 50, intrinsic viscosity 0.16–0.24 dL/g, and *M*_w_ = 7,000–17,000 Da), *O*-(2-aminoethyl)-*O'*-(2-carboxyethyl) polyethylene glycol hydrochloride (NH_2_-PEG-COOH, MW 3,000 Da), PFOB (CF_3_(CF_2_)_6_CF_2_Br; cat. no. 343862), *N*-hydroxysulfosuccinimide (sulfo-NHS), hydroxypropyl-beta-cyclodextrin (HP*β*CD), and 2-(*N*-morpholino)ethane sulfonic acid (MES) buffer were purchased from Sigma-Aldrich (St. Louis, MO). 1-Ethyl-3-(3-dimethylaminopropyl)carbodiimide hydrochloride (EDC) was purchased from Kanto Chemical (Tokyo, Japan). Methylene chloride (CH_2_Cl_2_, DCM), sodium cholate (SC), Nile Red, 4% paraformaldehyde, phosphate buffer saline (PBS), and other chemical reagents were laboratory grade and obtained from WAKO (Tokyo, Japan). Anti-EGFR antibody cetuximab (5 mg/mL) was provided by Merck KGaA (Darmstadt, Germany). RAW264.7, a murine leukemic macrophage line; MDA-MB-231, an EGFR-positive breast cancer cell line; and H520, an EGFR-negative lung cancer cell line, were obtained from ATCC (Rockville, MD). Cell culture media and reagents such as DMEM, phenol red–free RPMI 1640, fetal bovine serum (FBS), trypsin-EDTA solution were purchased from Gibco (Tokyo, Japan). Matrigel was from Corning Life Sciences (Tewksbury, MA). Female BALB/c nu/nu mice were from CLEA Japan, Inc., (Tokyo, Japan), and ddY mice were from Japan SLC (Hamamatsu, Japan). Reverse osmosis (RO) water was obtained using a RIOS/Synergy system from Merck Millipore (Billerica, MA). PLGA-*block*-PEG-COOH was synthesized by conjugation of PLGA-COOH to NH_2_-PEG-COOH following the previous method [22]. In the subsequent part of this article, PLGA and PLGA-PEG are referred to as PLGA-COOH and PLGA-*block*-PEG-COOH, respectively.

### 2.2. Nanocapsules Preparation

Nanocapsules were produced by the emulsion evaporation technique with slight modification [17]. A total mass of 100 mg polymer blend of PLGA and PLGA-PEG along with 60 *μ*L PFOB and 100 *µ*L (0.06 mg/mL) Nile Red dye was entirely dissolved in 4 mL DCM in a 20°C bath. The effect of PLGA-PEG amount to phagocytosis hindrance was evaluated by varying the PLGA to PLGA-PEG mass ratio in the initial formulation to 1 : 0 (**NC0%**), 9 : 1 (**NC10%**), 4 : 1 (**NC20%**), 3 : 2 (**NC40%**), 2 : 3 (**NC60%**), and 1 : 4 (**NC80%**) while keeping the total polymer amount constant (100 mg). In an attempt to produce nanocapsule with thinner shell, the total polymer mass in the formulation was reduced from the standard formulation of 100 mg (**NCm100**) to 40 mg (**NCm40**) and 20 mg (**NCm20**) with PLGA to PLGA-PEG mass ratio of 3 : 2, and PFOB amount remained constant.

Emulsification was performed in a 50 mL beaker immersed in ice. The organic phase was poured into 20 mL of 1.5% SC (w/v) and emulsified at 11,000 rpm by tissue homogenizer (Polytron, Kinematica, Luzern, Switzerland) for 30 s, continued with 30,000 rpm for 1 min. An ultrasonication with vibrating metallic tip (VC750, Sonics & Materials, Inc., Newton, CT; output 20 kHz, 750 W) was carried out for 1 min at 40% amplitude (focused energy input equals to ± 27–29 W/s). The emulsion was stirred for 4 h (300 rpm) at 20°C for complete solvent evaporation. The suspension was then syringe filtered on 0.8 *μ*m surfactant-free cellulose acetate Minisart NML filter (Sartorius, Goettingen, Germany) and ultrafiltered with 300 K MWCO PES Vivaspin 20 (Sartorius) at 1,000 g (4°C) for SC removal before being finally resuspended in 0.1 *μ*m-filtered RO water at a final concentration of about 60 mg/mL. For immediate characterization, fresh nanocapsule suspensions were kept in 4°C; while for extended storage, HPβCD (5% final concentration) was added before 48 h lyophilization (VD-550R, Taitec, Koshigaya, Japan).

### 2.3. Functionalization with Antibody

**Figure 1:**
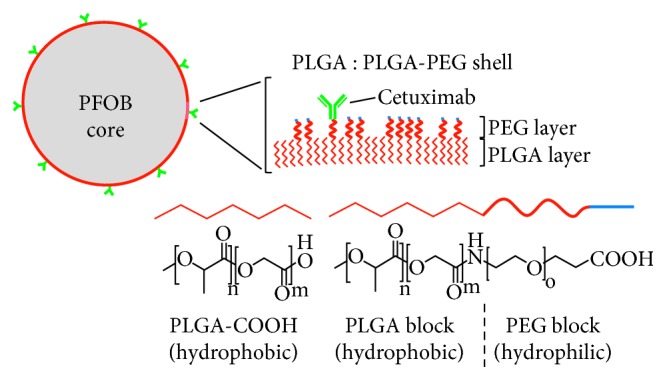
Schematic illustration of PFOB nanocapsules.

Nanocapsules (NCm100) were functionalized with cetuximab (5 mg/mL) via sulfo-NHS/EDC chemistry to produce cetuximab-labeled NCm100 following the previous method [23]. Basically, 10 mg nanocapsules (after buffer exchanged into 10 mM MES buffer; pH 5.5) were allowed to react with ultrapure water-dissolved sulfo-NHS (20 mg/mL, 12 *µ*L) and EDC (25 mg/mL, 3.06 *µ*L) in a conical glass vial at 4°C under magnetic stirring for 2 h to obtain nanocapsule sulfo-NHS ester. The reaction mixture was ultrafiltered (3,000 g; 4°C) through Amicon Ultra 0.5 mL 100 K MWCO (Merck Millipore, Merck KGaA, Darmstadt, Germany) at least three times with 10 mM MES pH 5.5 and two times with 10 mM PBS pH 7.4. The purified nanocapsule sulfo-NHS ester was then allowed to react with 1 mg cetuximab (after buffer exchanged into 10 mM PBS pH 7.4) in another conical glass vial at 4°C under magnetic stirring overnight. Final purification was performed by ultrafiltration (3,000 g; 4°C) through Amicon Ultra 0.5 mL 100 K MWCO at least five times with 10 mM PBS pH 7.4. The filtrate was collected and concentrated (ultrafiltration with 30 K MWCO filter) for quantification of unconjugated cetuximab using spectrophotometer (NanoDrop ND-1000, Thermo Fisher Scientific, Waltham, MA) at 280 nm UV absorbance. The cetuximab-labeling efficiency was calculated by comparing the proportion between the difference in cetuximab initial mixture concentration and after-labeling concentration, to the cetuximab initial mixture concentration. Cetuximab-labeled nanocapsules (cetuximab-labeled NCm100) were then lyophilized for storage. For *in vivo* experiments, saline-reconstituted cetuximab-labeled NCm100 and nonlabeled NCm100 were further prepared with filtration and short bath sonication (Bransonic CPX2800H, output: 40 kHz, 110 W) before use. Schematic illustration of PFOB nanocapsule is shown in Figure 1.

### 2.4. Nanocapsules Characterization

All nanocapsules characterization was performed after lyophilization, except cetuximab labeling efficiency calculation and electron microscopy observations.

#### 2.4.1. Size Distribution and Zeta Potential

Lyophilized nanocapsules were reconstituted in RO water (0.01 mg/mL). The hydrodynamic diameter (*d*_H_), polydispersity index (PDI), and zeta potential (ζ, surface charge) were measured in triplicate by dynamic light scattering (DLS) method for 60 s at 25°C and a 173° scattering angle using a Zetasizer Nano ZS (Malvern Instruments, Malvern, UK).

#### 2.4.2. Cryogenic Transmission Electron Microscopy (Cryo-TEM)

Cryogenic transmission electron microscopy was conducted at the JEOL Ltd. Laboratory (Tokyo, Japan). Fresh nanocapsule suspension (5 mg/mL) was deposited on a holey carbon film-coated R2/2 copper grids (Quantifoil Micro Tools GmbH, Jena, Germany) in the automatic plunge freezer system Leica EM GP (Leica, Wetzlar, Germany). The excess of solution was blotted off for 2 s, and the grids were snap-frozen in liquid ethane under a 90% humidity atmosphere. Samples were observed on a JEOL JEM-F200(CR) electron microscope operating at 200 kV. Images were acquired by a OneView camera (Gatan, Pleasanton, CA).

#### 2.4.3. Transmission Electron Microscopy (TEM)

Fresh nanocapsule suspensions (5 mg/mL) were dropped on a formwar film-covered copper grid (400 mesh) for 3 min. Grids with unstained samples were air-dried overnight before TEM observation. Negative staining was done by adding 40 *µ*L 4% uranyl acetate upon the samples on the grid, and excess solution was blotted off immediately with filter paper. Samples were observed on a JEM-1010 (JEOL, Tokyo, Japan) electron microscope operating at 80 kV. Images were acquired by a Veleta camera (Seika Digital Image, Tokyo, Japan).

#### 2.4.4. Shell Thickness Evaluation

Electron microscopy images were observed on Fiji software [24]. All measurable PFOB nanocapsules were evaluated. The ellipse selection tool was used to manually draw a region of interest (ROI) by encircling inner and outer edges of each nanocapsule shell to measure the PFOB core diameter (*d*_1_) and nanocapsule diameter (*d*_2_). Mean diameters were calculated by drawing two perpendicular lines dividing the ellipse or circular ROI. The T/R ratio was calculated as T/R ratio = (mean *d*_2_ – mean *d*_1_) ÷ mean *d*_2_.

#### 2.4.5. *In Vitro* Phagocytosis Study

A serial culture of RAW264.7 macrophage cell in 12-well plates (2–4 × 10^5^ cells/well, *n*=3) was incubated with 1 mg/mL nanocapsule solution of various PLGA-PEG amounts (NC0%-80%) in phenol red–free RPMI medium for 2.5 h at 37°C or 4°C. After PBS wash, cells were harvested, and nanocapsule uptake was evaluated in Attune Acoustic Focusing Flow Cytometer (Thermo Fisher Scientific, Waltham, MA) with RL-1 channel detected the Nile Red intensity. Nanocapsule uptake was evaluated as an increase in mean fluorescence intensity (MFI) in comparison with controls' autofluorescence. Each experiment was replicated three times.

#### 2.4.6. *In Vitro* EGFR-Targeting Study

MDA-MB-231 and H520 cells were cultured in 12-well plates, 2–4 × 10^5^ cells/well, in phenol red-free RPMI medium. Tumor targeting property of cetuximab-labeled NCm100 was evaluated by 2.5 h incubation of 0.77 mg/well of cetuximab-labeled NCm100 or NCm100, at either 37°C or 4°C. After PBS wash and fixation with 4% paraformaldehyde, cell nuclei were stained with 1 : 5,000 DAPI-PBS solution. Fluorescence microscopy observation (BZ-X700, Keyence, Osaka, Japan) was performed, and mean fluorescence intensity was used to measure the nanocapsule uptake.

### 2.5. Evaluation of PFOB Nanocapsule Echogenicity

Ultrasound study in ddY mice evaluated the nanocapsule echogenicity in subcutaneous injection lumps as well as in blood vessels and tumors after intravenous (i.v.) injection. Clinical ultrasonography system (Toshiba Aplio SSA-770A, Toshiba Medical System Corp., Otawara, Japan) and 7.5 MHz linear probe were used between 0.05 and 1.3 mechanical index (MI) [25], in differential tissue harmonic imaging (diff-THI) mode and advanced dynamic flow (ADF) contrast mode. Main MI tested in contrast mode was 0.2; however, after scan images with 0.2 MI were obtained, exploratory scans up to 0.7 MI were taken. Dynamic range and brightness was set at the beginning and kept untouched until the end of the study. The probe was placed in a modular fixation tool to ensure repeatability. Focus point was maintained as close as possible to the lowest base of targets (lumps, vessels, and tumors). Animal experiments were carried out following the Animal Facility guidelines and were approved by Animal Experiment Committee.

#### 2.5.1. Echogenicity Evaluation in Subcutaneous Lumps

Under maintained 2% isoflurane anesthesia, the mouse was positioned on her left side on a warm pad. Lumps were made by subcutaneously injecting 100 *μ*L of 2, 10, 25, or 50 mg/mL NCm100 on an imaginary line in a coronal plane on the right side (thorax to abdomen) to allow a side-by-side comparison between two or three lumps or with the tumors. Imaging plane of the linear probe was placed in the coronal plane to cover these subcutaneous lumps simultaneously.

#### 2.5.2. Echogenicity Evaluation in Blood Vessels

Under maintained anesthesia, the mouse was positioned supine on a warm pad. An i.v. dose of 25 or 50 mg/mL nonlabeled NCm100 (200 *μ*L) was slowly injected using a 27G syringe (10 s duration) via the tail vein. The linear probe was positioned to visualize liver and inferior vena cava (IVC) including other abdominal blood vessels along the transversal plane.

### 2.6. Evaluation of Nanocapsule EGFR-Targeting Property

#### 2.6.1. Tumor Xenografts

MDA-MB-231 and H520 tumor cells (10^6^ cells in 100 *μ*L 1 : 1 PBS-Matrigel solution) were implanted subcutaneously into the right flank of the nude mice (*n*=4 each) and grown for six weeks to reach 50 mm^3^ sizes. Tumor models were designed as small as possible to avoid necrotic formation in its center, yet large enough for detection under clinical ultrasound probe. The MDA-MB-231 tumor is EGFR-positive with poor and patchy vascularity when implanted subcutaneously [26]. Conversely, H520 tumor is EGFR-negative with dense, homogeneous vascular network [27].

#### 2.6.2. In Vivo and Ex Vivo Evaluation

Xenograft-bearing mice were i.v. injected via tail vein with 200 *μ*L of either 50 mg/mL cetuximab-labeled NCm100 or 50 mg/mL nonlabeled NCm100 (NCm100 or NC40%; dH = 120 nm). Under maintained 2% isoflurane anesthesia (1 L/min air flow; 5 min induction with 5%), the mouse was laid on her left side on a warm pad. Imaging plane of the linear probe was placed in the coronal plane to cover these tumors. Four imaging sessions were recorded. (1) *Baseline.* Static scan before injection, on both B-mode (1.0 MI) and contrast mode (0.2 MI), at the area comprising the largest part of the tumor. (2) *Injection session*. 10 s before injection followed by 50 s during and after injection. (3) *Postinjection session.* Static scan every 2 min for several minutes after injection. (4) *Late session.* 8 h, 15 h, and 24 h after injection. After the last ultrasound imaging, mice were sacrificed, and their tumors were collected.

The tumor was embedded in the optimum cutting temperature compound in a mold and snap frozen on liquid nitrogen vapor for cryosection (4 *μ*m). Tumor sections were prepared for fluorescence microscopy of cetuximab-labeled NCm100 and nonlabeled NCm100 visualization. DAPI staining was performed by Vectashield with DAPI (Vector Laboratories, Burlingame, California) to mark the cells nuclei. Observations were conducted under BZ-X700 fluorescence microscopy (Keyence, Osaka, Japan).

#### 2.6.3. Statistical Analysis

Data were expressed as the mean ± standard deviation (SD). Student's *t*-test was done to evaluate differences, which were considered significant at a *p* value of <0.05.

## 3. Results

### 3.1. Influence of PLGA-PEG Percentage to PFOB Nanocapsule Characteristics

**Figure 2:**
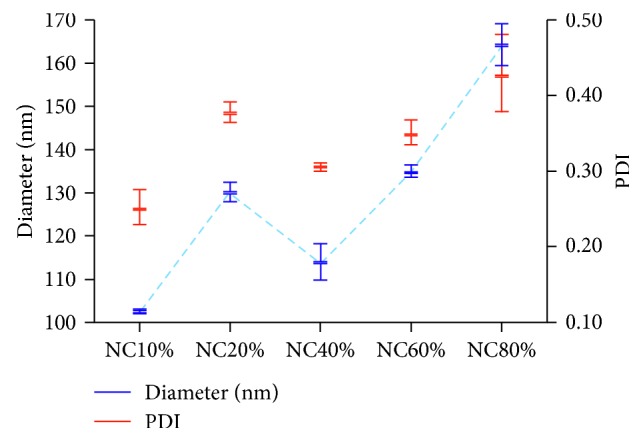
Mean diameters and polydispersity indices of PFOB nanocapsule prepared with different PLGA-PEG percentage (DLS measurement, *n*=3, error bars represent range).

Table 1 (upper row) compiles the characteristics of PFOB nanocapsules made with the various amount of PLGA-PEG.  Figure 2 shows the rising tendency of diameter and PDI along with the rise of PLGA-PEG percentage in the formulation. PFOB encapsulation was maintained in all nanocapsules formulation regardless the PLGA-PEG percentage (Supplementary Figure S1).

### 3.2. Influence of PLGA-PEG Percentage to Phagocytosis Hindrance Capability

**Figure 3:**
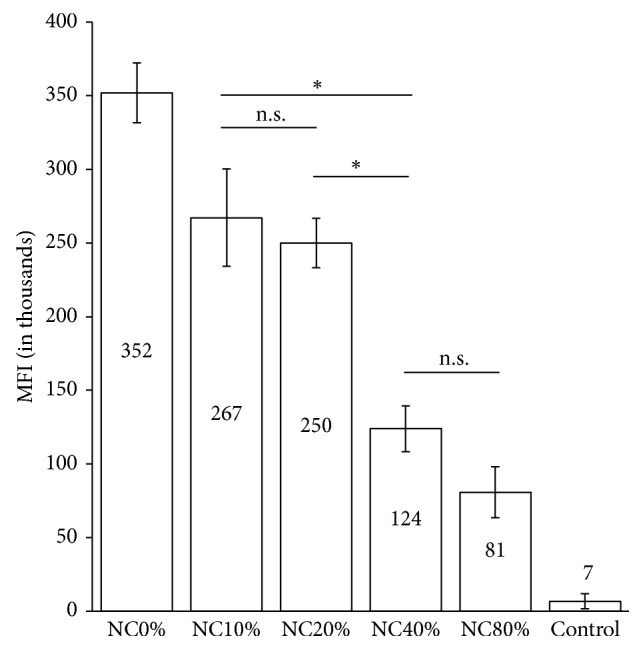
Phagocytosis uptake of nanocapsule with various amount of PLGA-PEG polymers. MFI: mean fluorescence intensity, ^*∗*^ *p* < 0.05; n.s., not significant.

At physiological temperature, NC0% was the most phagocytosed **(** Figure 3 **)**. NC40% demonstrated stronger phagocytosis hindrance than PFOB nanocapsules with lower PLGA-PEG percentages, while higher PLGA-PEG percentages did not significantly increase this capability. At 4°C, all PFOB nanocapsules (NC0% to NC80%) were phagocytosed at the similarly low amount (Supplementary Figure S2). Based on NC40% phagocytosis hindrance capability and also the better size and dispersity compared with NC80%, 3 : 2 mass ratio of PLGA and PLGA-PEG was used as the standard in the subsequent experiments.

### 3.3. Influences of Total Polymer Amount Modulation to Characteristics and Shell Thickness

PFOB nanocapsule size (*d*_H_) decreased from approximately 150 nm to less than 100 nm when total polymer mass was reduced from 100 mg to 40 and 20 mg, as measured by the DLS method **(** Table 1; *middle row* **)**. Nanocapsules stability was maintained on the borderline limit as reflected by zeta potential (*ζ*) of around −30 mV. PDI was similar among the three formulations (all >0.20), suggested their heterogeneous size (polydisperse).

**Figure 4:**
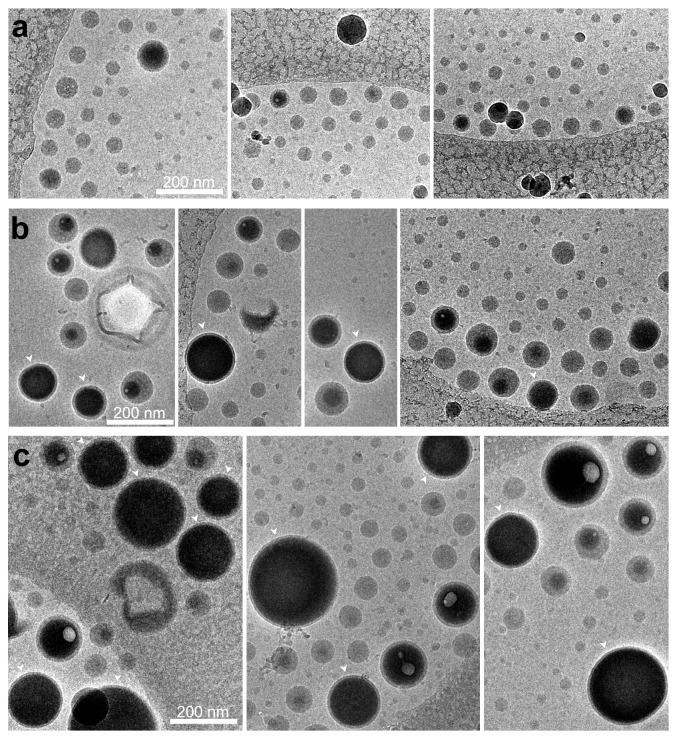
Representative cryo-TEM images of nanocapsule (PLGA: gray; PFOB: black) with various initial total polymer amount: (a) NCm20, (b) NCm40, and (c) NCm100. Nanocapsules with very thin shell are pointed with white arrowheads.

**Figure 5:**
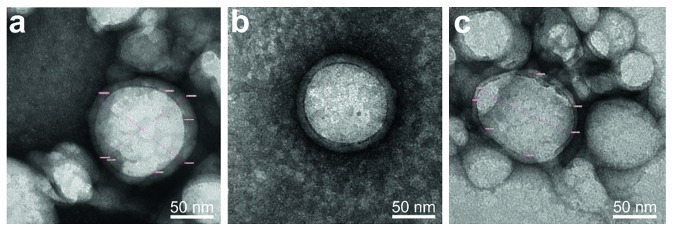
Representative negative-stained TEM images of nanocapsule (PLGA: dark gray; PFOB: light gray) with various initial total polymer amount: (a) NCm20, (b) NCm40, and (c) NCm100.

Cryo-TEM observation results confirmed these findings (Figure 4). Both cryo-TEM and negative staining TEM (Figure 5) revealed spherical nanostructures in all formulations. Their diameters were in a good agreement with the diameters measured by the DLS method. The population of the perfectly-formed core-shell nanostructure with centered PFOB core was higher in formulation with higher total polymer amounts, despite the solid polymer nanoparticles coexisted in all formulations (Figure 4). Negative staining improved the TEM image contrast and allowed visualization of inner and outer shell edges. However, accurate ROI drawing was not easily attainable even with maximum magnification.

**Figure 6:**
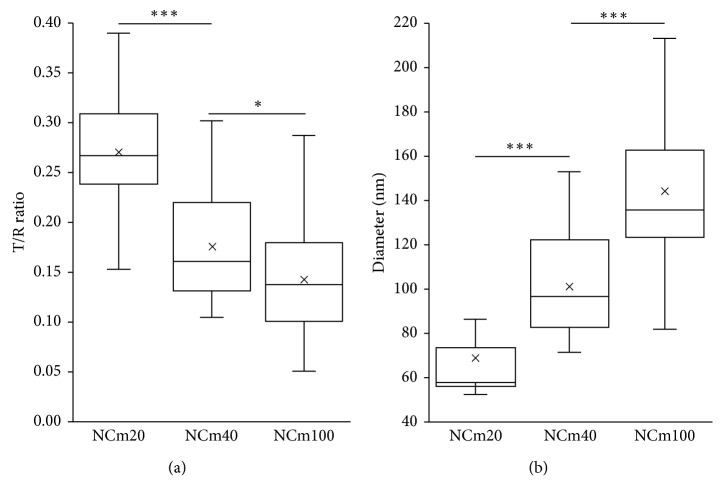
Distribution of (a) T/R ratio and (b) diameters of nanocapsule with various initial total polymer amount as measured from cryo-TEM images. ^*∗*^ *p* < 0.05, ^*∗∗∗*^ *p* < 0.0001.

Cryo-TEM images, on the other hand, clearly visualized well-defined shells in much higher resolution, thus allowed accurate measurement. Nanocapsules with very thin shells were observed in NCm40 and NCm100 samples (Figure 4 and 4). The mean T/R ratio of NCm100 and NCm40 was 0.146 ± 0.056 (*n*=75) and 0.178 ± 0.059 (*n*=28), respectively. The smallest achievable T/R ratio was 0.05 (NCm100). Unlike previous studies [4, 5], we found that reduction of the total polymer was unable to further decrease the T/R ratio (Figure 6(a)). PFOB nanocapsule size was reduced when total initial polymer amount is reduced (Figure 6(b)). Based on NCm100 small T/R achievement, 100 mg total polymer amount was used as the standard in the subsequent experiments.

### 3.4. Functionalization of PFOB Nanocapsules with Antibody

Cetuximab-labeled NCm100 was made with cetuximab labeling efficiency of approximately 90% based on quantification of unconjugated cetuximab. Table 1 (*lower row*) summarizes their final characteristics. The labeling process raised the cetuximab-labeled NCm100 size but did not drastically change the other characteristics. Preparation steps for *in vivo* studies, however, refined their PDI and zeta potential.

### 3.5. *In Vitro* Tumor-specific Targeting

**Figure 7:**
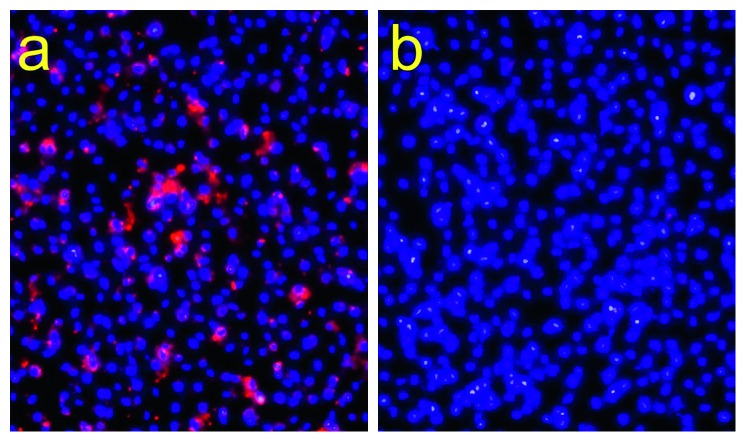
MDA-MB-231 cell uptake study of (a) cetuximab-labeled NCm100 and (b) nonlabeled NCm100 for specific targeting evaluation. Blue: nuclei (DAPI); red: cetuximab-labeled NCm100 or nonlabeled NCm100 (Nile Red).

At 37°C, cetuximab-labeled NCm100 was internalized into the cytoplasm of MDA-MB-231 cancer cells, while NCm100 was not (Figure 7). At the same temperature, neither cetuximab-labeled NCm100 nor NCm100 was internalized by H520 cancer cells (data not shown). At 4°C, both MDA-MB-231 and H520 had neither uptake of cetuximab-labeled NCm100 nor NCm100 (data not shown), validating the specific targeting capability of cetuximab-labeled NCm100.

### 3.6. Evaluation of Nanocapsule Echogenic Property

**Figure 8:**
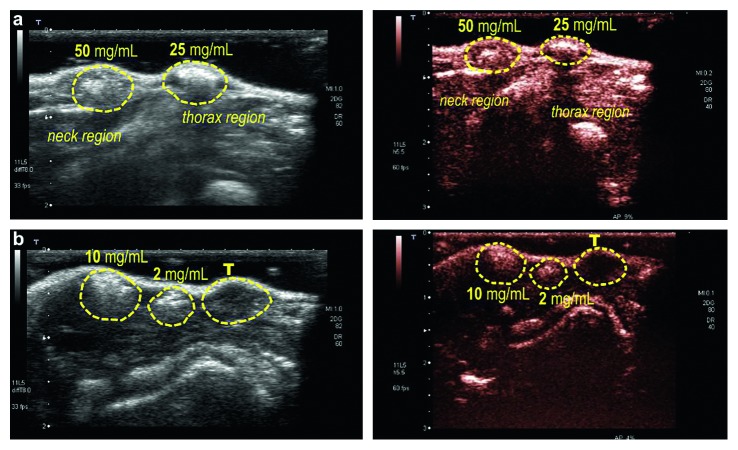
Echogenicity evaluation in subcutaneous lumps with two different concentrations of NCm100. THI-mode (left) and contrast mode (right) observation of two lumps with (a) high and (b) low concentrated NCm100. T, tumor.

**Figure 9:**
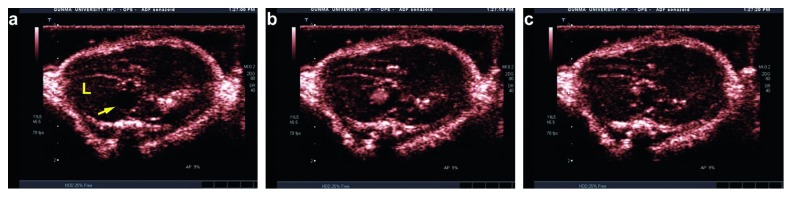
Echogenicity evaluation in the blood vessels. NCm100 enhanced inferior vena cava (yellow arrow) and disappeared within 22s (a) shortly before injection, (b) shortly after injection, and (c) 20s after injection. L, liver.

The subcutaneous lump of nonlabeled NCm100 showed an acoustic response at a dose as low as 2 mg/mL under ultrasound power as low as 0.1 MI in the contrast mode (Figure 8). Following the i.v. dose of 25 mg/mL, contrast enhancement was visible in IVC for about 20 s (Figure 9). Unexpectedly, no further contrast enhancement was observed in other abdominal blood vessels, nor in the hepatic parenchyma, even though the observation was continued until 20 min later. The injection of 50 mg/mL also did not prolong the IVC enhancement more than 30 s (data not shown).

### 3.7. Evaluation of Tumor-Specific Targeting

Following the i.v. injection of 50 mg/mL, contrast enhancements were not visible in both EGFR positive and EGFR negative tumors in all imaging sessions (Supplementary Figure S3). *Ex vivo* analysis showed that both nonlabeled NCm100 and cetuximab-labeled NCm100 have accumulated in the vascular area of the tumor regardless the availability of EGFR.

## 4. Discussion

This study aims to obtain thin-shelled PFOB nanocapsule from PLGA and PLGA-PEG blends, involving polymer reduction strategy. Phagocytosis hindrance, targeting capability, and acoustic response in *in vitro*/phantom and *in vivo* applications were also evaluated, once thin-shelled PFOB nanocapsules are obtained. Thin-shelled PFOB nanocapsule (T/R ratio = 0.15) was obtained from 3 : 2 mass ratio of 100 mg total polymer of PLGA and PLGA-PEG. However, polymer reduction strategy was unable to obtain further thinner shell. Phagocytosis hindrance, targeting capability, and acoustic response were adequate in *in vitro*/phantom study. The following passages will discuss the preparation toward the *in vivo* studies.

### 4.1. Increasing PLGA-PEG Percentage Raises the Size and Polydispersity

We found that all of our nanocapsules preserved spherical structure with PFOB core independently of PLGA-PEG percentage, as observed in microcapsule [17]. Similarly, the nanocapsule size tends to increase along with the raised proportion of PLGA-PEG. In microcapsules, PEG brush conformation determines the size expansion, since PFOB core remained uniform in all samples [17]. Our PFOB core heterogeneous size suggested that the nanocapsule size increase may not be affected only by the PLGA-PEG proportion.

PEGylated PLGA nanoparticles, prepared by similar emulsion evaporation method, also showed the similar size expansion tendency and larger than ours (160 nm for 10% PLGA-PEG; 200 nm for 80% PLGA-PEG) [28]. The emulsifier, polyvinyl alcohol (PVA), was speculated as the cause, due to its strong interaction with PEG. However, even though our emulsifier (SC) can be entirely removed during purification [16], the size expansion tendency remains. Size expansion also occurred when SC was used in the microcapsule preparation [17]. Therefore, other factors, not only emulsifiers, may contribute to this size expansion phenomenon, e.g., the differences between microcapsules and nanocapsules in (1) total energy input during emulsification and (2) their size order.

The increasing PDI following the increase of PLGA-PEG percentage indicates that our PFOB nanocapsules have a polydisperse size distribution. The higher inherent viscosity of PLGA-PEG may alter the phases equilibrium during evaporation, leading to size and PDI increase [28]. Therefore, if various percentages of PLGA-PEG are intended to formulate nanoparticles with similar size and PDI, the preparation process should be finely adjusted accordingly. However, the current PLGA: PLGA-PEG: PFOB nanocapsule system is more complicated than solid PLGA: PLGA-PEG nanoparticle. Such rigorous adjustment in emulsification step for PFOB nanocapsules warrants a further study.

### 4.2. PLGA-PEG Percentage Influences the Phagocytosis Hindrance Capability

The lack of discernible difference in phagocytosis hindrance between NC40% and NC80% in our study is likely due to the saturation of PEG brush conformation in NC80% as suggested in a previous study [28]. The surface of nanoparticles made of PLGA and PLGA-(15%) PEG is saturated at 1 : 1 PLGA : PLGA-PEG ratio (equal to NC50% in this study). Our nanocapsules were prepared from a custom-made PLGA-PEG copolymer with richer PEG amount, varies between 18 and 43%. Therefore, PEG saturation is expected when PLGA-PEG amount is above 40%. Theoretically, the use of SC, due to its lack of interaction with PEG, allows more PLGA-PEG addition before PEG brush density reaches saturation. Protein adsorption at nanoparticle surface, which lead to phagocytosis, achieves its optimum level when the distance between two terminal ends of PEG chains is about 1.4 nm [29]. However, a quantitative measurement such as X-ray photoelectron spectroscopy is necessary to confirm whether such PEG density level already achieved at NC40% surface. Since phagocytosis hindrance capability of NC40% is not different from NC80%, and NC40% also has more favorable size and dispersity for tumor targeting, NC40% was used throughout the *in vivo* tests.

### 4.3. Total Polymer Amount Reduction Cannot Minimize the Shell Thickness

Polymer amount reduction produced thin-shelled PFOB nanocapsule from plain PLGA [4, 5]. However, PLGA-PEG copolymer [21] or other polymer blends [13] [14] did not promote shell thickness reduction using this strategy. When only PLGA-PEG is used, thick-shelled nanocapsules are formed along with PFOB globules [21]. We found that polymer reduction strategy, in our case of PLGA and PLGA-PEG blend, decreased the population of thin-shelled PFOB nanocapsules and increased the number of solid polymer nanoparticles (Figure 4). However, acorn, oblate, elongated, or tears of wine nanostructure were not observed, unlike when PLA was used [13, 15].

COOH-PEG moieties of PLA-PEG-COOH might be responsible for elongated shape [13] while COOH moieties of PLA-COOH produced decentered-core shape [15]. In our system, both PLGA and PLGA-PEG are also carboxylated. The COOH moieties of PLGA-PEG-COOH may be responsible for the formation of solid nanoparticle, thick-shell nanocapsules, and decentered PFOB-core. In future studies, optimation using a mixture of methoxy-terminated PLGA-PEG and PLGA-PEG-COOH might be one option to reduce the formation of decentered-core shape population while maintaining the functionalization potential.

On the other hand, COOH moieties of PLGA may help to increase PLGA adsorption by lowering its viscosity, leading to the formation of a thinner shell. As formerly known, PLGA maintains the surface tension difference between phases high enough to stabilize PFOB droplets inside the emulsion globules [16]. Our finding suggests that a certain PLGA amount is indispensable within formulation to produce enough thin-shelled PFOB nanocapsule population.

The mean T/R ratio of NCm100 and NCm40 was well below than that of standard PLGA PFOB nanocapsule (0.25–0.35) [4, 5]. However, the shell thickness was polydispersed within the same sample (Figures 4, 4, and 6(a)), as previously observed with PLGA-PEG [21]. Since NCm100 average diameter (145 ± 34 nm, Figure 6(b)) is similar to that of PLGA nanocapsules [4, 5], we expected an acoustic response improvement.

### 4.4. Thin-Shelled PFOB Nanocapsule Demonstrated Echogenicity in Low Concentration and Low Ultrasound Power

Our PFOB nanocapsule system (NCm100) demonstrated an acoustic response in low concentration, 2 mg/mL. This concentration is approximately equal to the final concentration of nanocapsule in blood circulation, considering the total mouse blood volume. In a previous report, visible contrast enhancement in IVC lasted only a few seconds, despite the high nanocapsule concentration (50 mg/mL) and high ultrasound power (MI 1.6) [4]. Our thin-shelled PFOB nanocapsules demonstrated contrast enhancement in IVC for about 20 s using a smaller dose and less ultrasound power. Even though the current PEGylated PFOB nanocapsule is relatively sensitive to low ultrasound power, in an *in vivo* scenario, these advantages might be insufficient due to several reasons. First, the final concentration might be diluted in circulation to less than 2 mg/mL. Thus, the advantage of the small T/R ratio may be deficient to generate an observable acoustic response. Second, the current stealth property might not yet be sufficient to protect the circulating amount of nanocapsule at a detectable level. Studies showed that even full PEGylation on nanoparticle surface remains unable to completely diminish liver and splenic trapping [18, 20, 22].

The latest theoretical model showed that thinner shell significantly raises echogenicity [10, 12]. Another model predicted a weak acoustic response of nanocapsules with PFOB core (75 nm and 150 nm) in dilute suspension (as low as 1% concentration) in which shell parameters are important (PLGA-PEG, at T/R ratio of 0.25) [10, 30]. Our current findings verify that shell thickness is a key factor for further modification to achieve a balance between echogenicity and reasonable particle size.

### 4.5. Evaluation of Tumor-specific Targeting

*In vitro* specific targeting to EGFR receptors has been demonstrated using cetuximab bound at the end of 3,000 Da PEG chains. The use of arbitrary dose of 1 mg cetuximab did not affect the stability and the stealthiness of cetuximab-labeled NCm100, supporting the specific targeting to the EGFR-positive cells only. The optimized PEGylation also helped minimize nonspecific uptake of NCm100. A large serial experiment may simultaneously evaluate the relation between PEG amounts in PFOB nanocapsule surface and antibody concentration for labeling, which currently lacking from our study due to the limited yield in PFOB nanocapsule preparation.

The main concerns for antibody-labeled nanoparticles are (1) the antibody size (∼15–20 nm) relative to the nanoparticle size (∼150 nm) and (2) the replacement of PEG, which might drastically affect nanoparticles' stealth property [31]. The absence of surface charge alteration in cetuximab-labeled NCm100 suggested that cetuximab labeling may not affect the PEG stealth property, as indicated in a previous review [18]. However, further investigation is necessary to obtain a more precise balance between the maximum PEG stealth capacity that remains and the minimum antibody amount to maintain specific targeting.

We are also interested whether specific targeting improves tumor accumulation and eventually increase the acoustic response. Our experiments using two distinct tumors regarding target availability and vascularity pattern demonstrated that tumor accumulation of antibody-labeled PFOB nanocapsules relied merely on EPR effect, inadequate to improve tumor accumulation. Active targeting of any particles will face tumor microenvironment challenges, such as blocking by interstitial collagen matrix [1, 32]. Collagen carries a positive charge, which may trap our negatively charged (±−40 mV) cetuximab-labeled NCm100. Additionally, nanoparticle larger than 60 nm may not effectively diffuse through the interstitial space [32]. While particle size is crucial for maintaining the acoustic response, a tiny nanocapsule (<60 nm) may require an extremely small T/R ratio to retain the shell compressibility for a detectable acoustic response [12]. Such extreme design indicates that modification in material and preparation remains a challenge.

Our study has several main limitations. First, PFOB encapsulation was not quantified. While more solid nanoparticles and PFOB nanocapsules with thicker shells were formed when the total polymer is reduced, we can only assume that PFOB encapsulation was reduced. Second, precise quantification of acoustic response was not performed. Such quantification will require another mechanical phantom for *in vitro* test and image processing tool from a dedicated contrast mode module for ultrasound system for animal study. Third, the phagocytosis was not evaluated in *in vivo* study, which may provide clues for the quick disappearance of contrast enhancement.

## 5. Conclusions

We have developed thin-shelled targeted PFOB nanocapsules from PLGA and PLGA-PEG blends that preserved both echogenicity and tumor-specific targeting. The 3 : 2 mass ratio of PLGA and PLGA-PEG yielded mean T/R ratio down to 0.15. Polymer reduction strategy was unable to further downscale the T/R ratio. A low concentration (2 mg/mL) of thin-shelled PFOB nanocapsules can be detected by low-power ultrasound (7.5 MHz, 0.2 MI), but this acoustic response might not yet adequate to support *in vivo* tumor detection with the current tumor accumulation level. This preliminary finding suggested that the shell and core material choice and the formulation of thin shell remains open for improvement.

## Figures and Tables

**Table 1:** Characteristics of PFOB nanocapsules from different formulations (DLS measurement, *n*=3 per sample).

NC	Mixing ratio		Total polymer mass (mg)	*d*_H_ (nm)	PDI	ζ (mV)
	PLGA (%)	PLGA-PEG (%)				
NC10%	90	10	100	102.5 ± 0.4	0.249 ± 0.02	−46.2 ± 1.7
NC20%	80	20	100	130.0 ± 2.3	0.377 ± 0.01	−42.2 ± 2.9
NC40%	60	40	100	113.9 ± 4.2	0.306 ± 0.01	−44.7 ± 2.2
NC60%	40	60	100	134.7 ± 1.6	0.348 ± 0.02	−47.0 ± 1.8
NC80%	20	80	100	164.2 ± 4.9	0.426 ± 0.05	−42.5 ± 3.6
NCm20	60	40	20	95.6 ± 7.7	0.394 ± 0.01	−30.1 ± 2.3
NCm40	60	40	40	87.0 ± 2.1	0.266 ± 0.03	−29.8 ± 2.4
NCm100	60	40	100	156.6 ± 5.7	0.385 ± 0.03	−27.7 ± 1.3
Nonlabeled NCm100^*∗*^	60	40	100	120.1 ± 2.6	0.229 ± 0.02	−61.4 ± 0.9
Nonlabeled NCm100^*∗∗*^				101.8 ± 0.3	0.157 ± 0.01	−39.3 ± 0.6
Cetuximab-labeled NCm100^*∗*^	60	40	100	234.1 ± 2.3	0.328 ± 0.03	−50.0 ± 1.2
Cetuximab-labeled NCm100^*∗∗*^				159.2 ± 1.8	0.183 ± 0.02	−41.0 ± 2.6

^*∗*^Before and ^*∗∗*^after being prepared for *in vivo* studies, by filtration and short sonication.

## Data Availability

The data used to support the findings of this study are available from the corresponding author upon request.

## Supplementary Materials

Supplementary Materials The three additional figures are important to further describe our result, but may not sufficient to be considered as main figures and placed along the text article.
