# Supplementary material for: Thin-Shelled PEGylated Perfluorooctyl Bromide Nanocapsules for Tumor-Targeted Ultrasound Contrast Agent
Source: Contrast Media Mol Imaging. 2018 Nov 1;2018:1725323. doi: 10.1155/2018/1725323 (PMC6236697; doi:10.1155/2018/1725323)

**SUPPLEMENTARY MATERIALS**

**Fig. S1** Representative unstained-TEM images of (a.) NC10%, (b.) NC20%, (c.) NC40%, (d.) NC60%, (e.) NC80%, and (f.) low magnification of NC40%. PFOB shown as grey shade encapsulated by PLGA (dark circles).


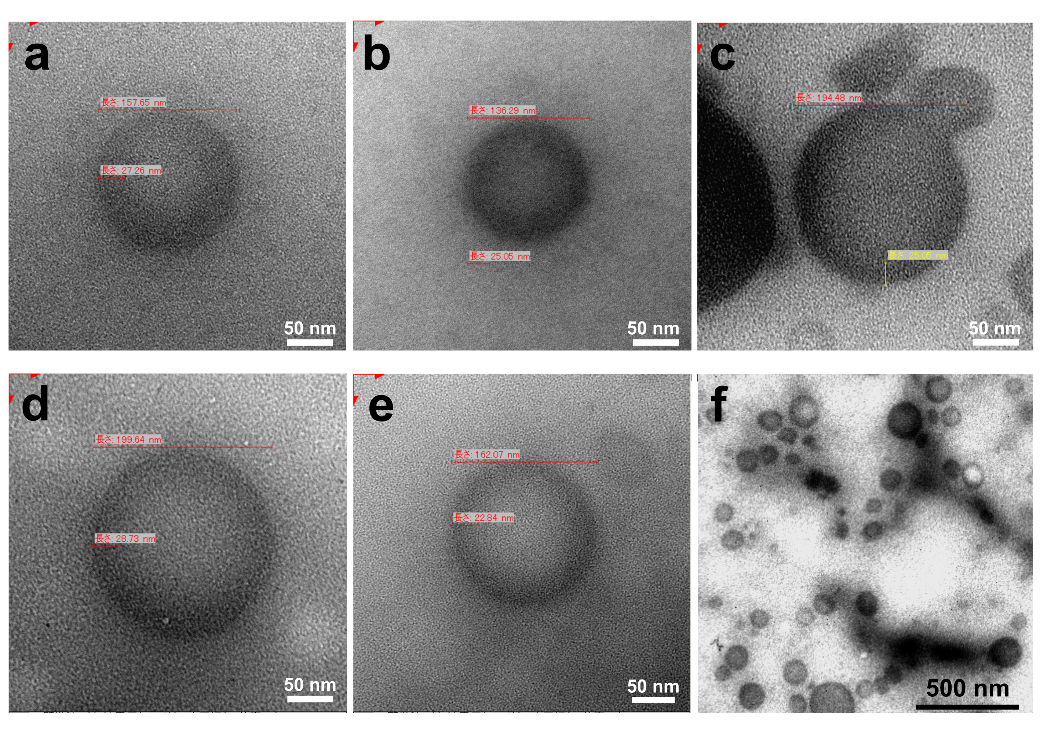


**Fig. S2** Flow cytometry histogram of phagocytosis study of PFOB nanocapsules with various PLGA-PEG amounts, at 4 ^o^C. For each sample, n = 3.


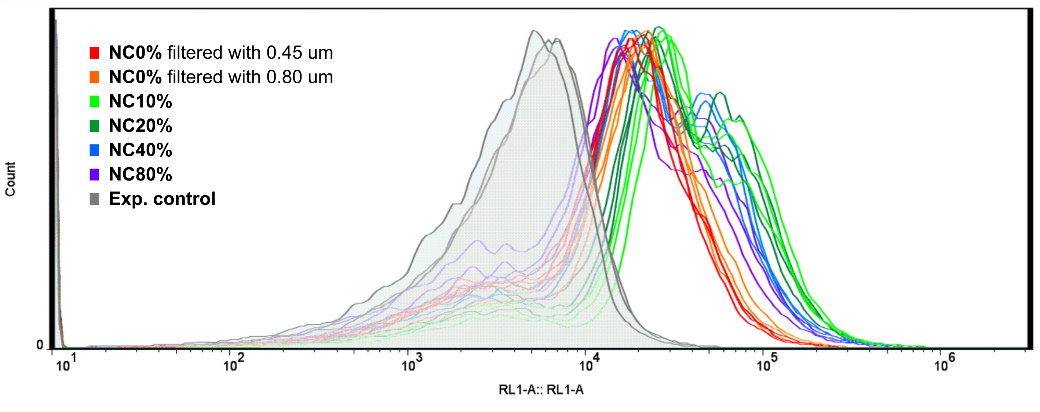


**Fig. S3** *In vivo* and *ex vivo* study for tumor-targeting evaluation after i.v. injection of 50 mg/mL cetuximab-labeled NCm100 or non-labeled NCm100 in MDA-MB-231 (EGFR-positive) and H520 (EGFR-negative) tumors. Tumors were sectioned following the tumor position in ultrasound imaging. ***First row:*** Ultrasound images on late sessions (24 h and 15 h postinjection for MDA-MB-231and H520, respectively). ***Second row:*** Tumor tissue macro sections. ***Third row:*** Fluorescence microscopy images, ***red:*** cetuximab-labeled NCm100s and non-labeled NCm100, ***blue:*** cell nuclei. ***Fourth row:*** Tumors involved in *in vivo* evaluation of specific targeting. H520 tumors, in particular, were more homogeneously hypervascular than MDA-MB-231. ***Abbreviations:*** **s.**: solid and dense parts, **v.**: hypervascular parts.


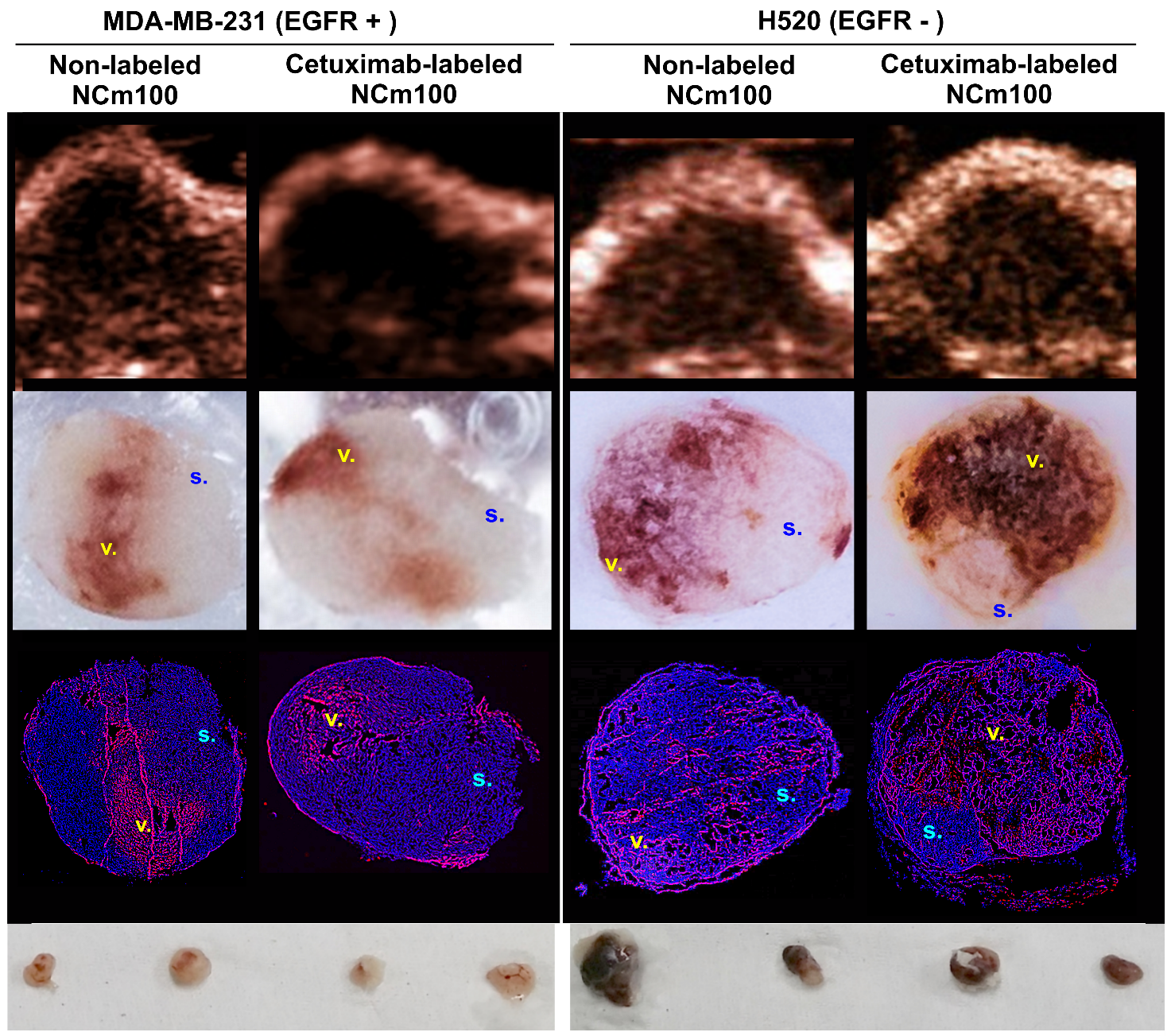

Supplement: Supplementary Materials — The three additional figures are important to further describe our result, but may not sufficient to be considered as main figures and placed along the text article. [file 1725323.f1.docx]
